# Supplementary material for: Relationships between objective structured clinical examination, computer-based testing, and clinical clerkship performance in Japanese medical students
Source: PLoS One. 2020 Mar 26;15(3):e0230792. doi: 10.1371/journal.pone.0230792 (PMC7098585; doi:10.1371/journal.pone.0230792)
Supplement: Attachment — Submitted filename: response to reviewers.doc [file pone.0230792.s002.doc]

**To the Editor (Prof. Conor Gilligan, M.D.)**

**Editor comments: This paper addresses an important topic in medical education but some further detail and justification is needed to strengthen the paper and ensure it makes a valuable contribution. Both reviewers make helpful points which you should take into consideration in preparing a revised version. In particular, I agree that the study needs clearer justification and I would think that further discussion of the theories associated with competency-based assessment and the challenges of teaching and assessment in clinical settings would be an important addition. Also, please provide further detail on the methods including the assessment measures and scoring systems. The discussion will need to change to reflect these changes to other sections.**

Thank you for appreciating the clinical importance regarding our article.

We revised the manuscript faithfully according to the insightful comments of the reviewers as possible, especially for justification and discussion on the theory on competency-based assessment and challenges of teaching and assessment. We believe that simulation-based education can be a solution for competency-based assessment and dissolving the problems associated with teaching and learning in clinical settings.

We believe that the quality of our article improved significantly by their valuable comments.

**To Reviewer 1**

Thank you for appreciating the educational significance of our article.

**1.  Reviewer comments;** **Introduction**

**The introduction lacks in depth discussion and information about the relationship between OSCE, written examination and Mini-CEX. The authors have provided the organizational context in the introduction but have failed to highlight what is already known in the literature about the relationship between OSCEs, written examination and Mini-CEX. Some examples of published literature include**

**•** **Susan Humphrey-Murto, Mylène Côté, Debra Pugh & Timothy J. Wood (2018) Assessing the Validity of a Multidisciplinary Mini-Clinical Evaluation Exercise, Teaching and Learning in Medicine, 30:2, 152-161, DOI: 10.1080/10401334.2017.1387553**

**• Rogausch A, Beyeler C, Montagne S, Jucker-Kupper P, Berendonk C, Huwendiek S, Gemperli A, Himmel W. The influence of students’ prior clinical skills and context characteristics on mini-CEX scores in clerkships–a multilevel analysis. BMC medical education. 2015 Dec;15(1):208.**

**Given that there are published evidence highlighting the relationship between the above variables, the authors need to identify the gap and state the justification for the study.**

**In addition, some of the references listed such as references, one (1) to four (4) do not reflect the statements written by the authors in the first paragraph. Please use appropriate references.**

Thank you for constructive comments regarding our article. According to the suggestion of Reviewer#1, we re-wrote the introduction part for justification. We also changed the references to appropriate ones.

**2.**  **Reviewer comments;** **Methods**

**Study measures**

**OSCE content and evaluation**

**The authors stated that seven aspects were covered in the OSCE. Could the authors state the clinical disciplines that were examined? Was it only one discipline or was the OSCE conducted in all disciplines (Internal Medicine, Pediatrics, Obs and Gyn, Surgery, Family medicine, Emergency, and Anesthesiology)?**

**How was the overall score calculated?**

**CBT Content and Evaluation**

**What content was covered? Were the clinical disciplines assessed? If so, please state it.**

**Clinical clerkship content and evaluation**

**The authors have stated that an evaluation sheet based on Mini-CEX and DOPS was used. Could the authors include a copy of the evaluation sheet?**

**In addition, the authors have stated that the basic clinical clerkship was conducted across all disciplines. How was the average and overall score for the mini-CEX calculated as well as the scores for the different clinical disciplines? Was the form adapted for the different clinical disciplines? If so, please provide the details**

**Statistical analysis**

**The statistical analysis should be updated based on the information provided above related to the methods.**

Thank you for constructive comments regarding our article. According to the suggestion of Reviewer#1, we changed our manuscript accordingly.

1, We apologize for the expression. We performed this basic OSCE which includes basic OSCE which is common to all disciples. The seven parts is chest examination, abdominal examination, head and neck examination, neurological examination, emergency response, basic technique which we evaluated in the OSCE. We added this information clearly.

2, Over all score was calculated of the average of the seven OSCE stations.

3, CBT included basic clinical disciplines and basic medicine knowledges.

4, We also added the summary of clinical clerkship evaluation sheet as Figure 2. We made this sheet which we can make scoring and evaluation definitely.

**3, Reviewer comments; Results**

**As stated in the methods section, the authors need to provide further information on how the average score reported were calculated for the OSCE, CBT and CC. This applies to all sections of the results. Given the above issues, the results reported by the authors cannot be verified.**

Thank you for constructive comments regarding our article. According to the suggestion of Reviewer#1, we clarified the average score calculation method. We again checked our statistics method and confirmed the correct results.

**4, Reviewer comments; Discussion**

**Given the concerns raised about the methodology and results, the discussion needs to be re-written to align with the updated information.**

**However, the first paragraph in the discussion is a repetition of the first paragraph in the introduction. The authors need to consider how to present the findings of the study in relation to existing evidence.**

Thank you for constructive comments regarding our article. According to the suggestion of Reviewer#1, we re-wrote the discussion part.

We sincerely appreciate for your insightful suggestions regarding our article. We believe that our manuscript has been significantly improved by your valuable comments.

**To Reviewer 2**

Thank you very much for appreciating the educational significance of our article.

We all agree with all your insightful comments and revised the manuscript accordingly.

**1. Reviewer comment; It would be good to know what teaching was done prior to the test. Did all 94 students attend the session. was it mandatory? was it right before the test or was there a lag period? This was not clear on the manuscript and would have a significant impact on the results. Please clarify.**

Thank you for constructive comments regarding our article. According to the suggestion of Reviewer#2, we added the concise information of the OSCE evaluation in the introduction and method part.

**2. Reviewer comment; What elements of the examination (head and neck exam; neurological examination etc.) was expected in the five minute OSCE period. So, specifically for the chest exam, was it just the preacordium that was examined in the five minutes? For neurological exam was it all aspects of sensation, proprioception, reflexes for upper and lower limb that was done in the five minutes? Please clarify.**

Thank you for constructive comments regarding our article. According to the suggestion of Reviewer#2, we added more information about the OSCE. Actually, the scenario theme was randomly selected and medical students perform core examination skill in each part. We added this in the method part.

**3. Reviewer comment; Did the 2 examiners scores correlate? An average was achieved. Was examiner training provided to ensure consistency?**

Thank you for constructive comments regarding our article. We confirmed that 2 examiners scores are correlated. Also, we perform 3 hour evaluation training before OSCE. According to the suggestion of Reviewer#2, we added some training for OSCE evaluation.

**4. Reviewer comment; Typo in paragraph 2 of study measures. 'the examination also strictly checks the identification of students.....'**

Thank you for constructive comments regarding our article. According to the suggestion of Reviewer#2, we corrected the expression.

**5. Reviewer comment; For the computer based testing, were the 320 question standard tested beforehand? If so, was this to the standard of a fourth-year or a 5th year medical student?**

Thank you for constructive comments regarding our article. This is a standard for 4th-year student. According to the suggestion of Reviewer#2, we added this in the introduction part.

**6. Reviewer comment; In addition clarification should be provided as to what aspects of clinical knowledge was tested. Was it all subjects or just acute medicine and acute surgery that is applied during clinical clerkship?**

Thank you for constructive comments regarding our article. The CBT includes basic clinical knowledge in all disciplines. According to the suggestion of Reviewer#2, we added this information in the method part.

**7. Reviewer comment; It looks like the mini clinical evaluation exercise and the direct observation of procedural skills assessement tools were graded for correlation purposes. Could the grading/marksheets be provided, please. Traditionally these assessments are used for feedback purposes and not usually graded.**

Thank you for constructive comments regarding our article. According to the suggestion of Reviewer#2, we added the clinical clerkship grading sheet as Figure 2. However, OSCE evaluation sheer contain some restrictions by Common Achievement Test Organization in Japan. We added the evaluation standards of OSCE in the methods part as possible with citation.

We sincerely appreciate for your insightful suggestions regarding our article. We believe that our manuscript has been significantly improved by your valuable comments.
